# Evaluating the dosimetric impact of deep‐learning‐based auto‐segmentation in prostate cancer radiotherapy: Insights into real‐world clinical implementation and inter‐observer variability

**DOI:** 10.1002/acm2.14569

**Published:** 2024-12-01

**Authors:** Najmeh Arjmandi, Mohammad Amin Mosleh‐Shirazi, Shokoufeh Mohebbi, Shahrokh Nasseri, Alireza Mehdizadeh, Zohreh Pishevar, Sare Hosseini, Amin Amiri Tehranizadeh, Mehdi Momennezhad

**Affiliations:** ^1^ Department of Medical Physics Faculty of Medicine Mashhad University of Medical Sciences Mashhad Iran; ^2^ Physics Unit, Department of Radio‐Oncology Shiraz University of Medical Sciences Shiraz Iran; ^3^ Ionizing and Non‐Ionizing Radiation Protection Research Center School of Paramedical Sciences Shiraz University of Medical Sciences Shiraz Iran; ^4^ Medical Physics Department Reza Radiation Oncology Center Mashhad Iran; ^5^ Medical Physics Research Center Faculty of Medicine Mashhad University of Medical Sciences Mashhad Iran; ^6^ Department of Radiation Oncology Mashhad University of Medical Sciences Mashhad Iran; ^7^ Cancer Research Center Mashhad University of Medical Sciences Mashhad Iran; ^8^ Department of Medical Informatics Faculty of Medicine Mashhad University of Medical Sciences Mashhad Iran

**Keywords:** auto‐contouring, deep learning, inter‐observer variability, prostate segmentation, radiotherapy treatment planning

## Abstract

**Purpose:**

This study aimed to investigate the dosimetric impact of deep‐learning‐based auto‐contouring for clinical target volume (CTV) and organs at risk (OARs) delineation in prostate cancer radiotherapy planning. Additionally, we compared the geometric accuracy of auto‐contouring system to the variability observed between human experts.

**Methods:**

We evaluated 28 planning CT volumes, each with three contour sets: reference original contours (OC), auto‐segmented contours (AC), and expert‐defined manual contours (EC). We generated 3D‐CRT and intensity‐modulated radiation therapy (IMRT) plans for each contour set and compared their dosimetric characteristics using dose‐volume histograms (DVHs), homogeneity index (HI), conformity index (CI), and gamma pass rate (3%/3 mm).

**Results:**

The geometric differences between automated contours and both their original manual reference contours and a second set of manually generated contours are smaller than the differences between two manually contoured sets for bladder, right femoral head (RFH), and left femoral head (LFH) structures. Furthermore, dose distribution accuracy using planning target volumes (PTVs) derived from automatically contoured CTVs and auto‐contoured OARs demonstrated consistency with plans based on reference contours across all evaluated cases for both 3D‐CRT and IMRT plans. For example, in IMRT plans, the average *D*_95_ for PTVs was 77.71 ± 0.53 Gy for EC plans, 77.58 ± 0.69 Gy for OC plans, and 77.62 ± 0.38 Gy for AC plans. Automated contouring significantly reduced contouring time, averaging 0.53 ± 0.08 min compared to 24.9 ± 4.5 min for manual delineation.

**Conclusion:**

Our automated contouring system can reduce inter‐expert variability and achieve dosimetric accuracy comparable to gold standard reference contours, highlighting its potential for streamlining clinical workflows. The quantitative analysis revealed no consistent trend of increasing or decreasing PTVs derived from automatically contoured CTVs and OAR doses due to automated contours, indicating minimal impact on treatment outcomes. These findings support the clinical feasibility of utilizing our deep‐learning‐based auto‐contouring model for prostate cancer radiotherapy planning.

## 1 INTRODUCTION

Accurate definition of the clinical target volume (CTV) and organs at risk (OARs) is crucial for successful cancer radiotherapy. These contours directly impact treatment planning, affecting tumor control and complications in healthy tissues.^1^, ^2^ However, manual contouring can be challenging, especially with male pelvic CT images, where low soft tissue contrast and variable organ shapes pose difficulties.^3^, ^4^ Accurate delineation of the prostate CTV, along with OARs like the bladder, rectum, and femoral heads, is crucial for effective prostate cancer treatment.^5^, ^6^ These contours are used to optimize and evaluate the quality of treatment plans.^6^ Recently, many treatment planning software vendors have integrated deep‐learning‐based auto‐contouring to streamline this process.

Auto‐contouring techniques offer several advantages in clinical practice. They significantly reduce inter‐ and intra‐observer variability, leading to more consistent and reliable contouring. By automating the process, these techniques alleviate workload for radiation oncologists, thus accelerating the treatment planning process.^7^, ^8^, ^9^

While numerous studies have shown promising results for automatic contouring of multiple organs in the male pelvis for prostate cancer patients, these studies have primarily focused on geometric evaluation^5^, ^10^, ^11^, ^12^, ^13^ Implementing auto‐contours in real‐world radiotherapy treatment planning requires further evaluation beyond geometry. Dosimetric assessment of automatic contours is important for successful integration, as geometric evaluation alone is insufficient for optimal utilization in treatment planning.^2^, ^9^, ^14^

Several studies have demonstrated high levels of geometric and dosimetric agreement between automated and manually contoured structures, suggesting the potential for clinically acceptable dose distributions. Kaderka et al., for instance, demonstrated high geometric and dosimetric concordance, suggesting that auto‐contouring can generate dose distributions comparable to those achieved through manual delineation.^15^ This observation is supported by Van Dijk et al., who found that employing higher‐precision auto‐contouring algorithms significantly reduced dosimetric discrepancies.^16^ Furthermore, Guo et al. demonstrated minimal impact on most dose‐volume metrics when using automated contours.^9^

However, despite often achieving high geometric accuracy, Numerous studies have identified significant limitations in the dosimetric accuracy of automated contouring. Sritharan et al. observed instances of suboptimal target coverage with automated contours, underscoring the risk of inadequate treatment delivery if manual review is omitted.^4^ These findings are further supported by Kawula et al.,^2^ who reported dose distribution discrepancies, particularly pronounced at organ boundaries, and Starke et al.,^17^ who demonstrated cases of both suboptimal target coverage and inaccurate organ doses resulting from the use of unedited auto‐contours.

These studies suggest that while deep learning models can accurately segment organs, dosimetric evaluation is crucial for their optimal implementation in radiotherapy treatment planning. Dosimetric evaluation of deep‐learning‐based automatic segmentation in prostate cancer on CT images remains under‐investigated, highlighting a need for more comprehensive studies to assess its clinical impact. Furthermore, while it is often claimed that deep learning reduces inter‐observer variability,^18^, ^19^, ^20^, ^21^ the impact of this technology on this aspect remains under‐explored. Robust comparisons with manual contouring by multiple observers are crucial to definitively assess the contribution of deep learning to consistency in treatment planning.

In our previous work,^22^ we evaluated the geometric impact of auto‐segmentation contour models for CTV and OARs in prostate cancer radiotherapy. However, we did not assess the practical applicability of these auto‐contours. This paper takes a step further by evaluating the effectiveness of using auto‐contours for CTV and OAR delineation in actual prostate cancer radiotherapy treatment planning. We aim to evaluate the dosimetric impact of deep‐learning‐based auto‐contoured organs compared to the gold standard manual contours. This involves analyzing the dose distributions and evaluating the potential advantages and disadvantages of using auto‐segmentation in clinical practice. We also investigated the inter‐expert variability in planning CT scans, comparing the accuracy and consistency of auto‐contouring system to the inherent variability observed between human experts.

## 2 METHODS AND MATERIALS

### 2.1 Auto‐contouring system

We previously developed an in house deep‐learning‐based auto‐contouring method for segmenting the CTV and OARs in prostate cancer patients.^22^ This method utilizes 104 retrospective planning CT scans from patients with localized prostate cancer who underwent external beam radiotherapy. Our proposed hybrid CNN‐ViT model, VGG16‐UNet‐ViT, demonstrably outperforms current state‐of‐the‐art methods^2^, ^14^, ^17^, ^21^ in generating precise contours for prostate segmentation. The model leverages VGG16 as a CNN encoder and incorporates a novel fusion block to integrate global and spatial features from both ViT and CNN encoders. An ablation study was conducted to assess the contributions of CNN and ViT components. The model was trained on 70% of the data, validated on 10%, and tested on the remaining 20%. Furthermore, the CT images were sourced from multiple centers with diverse patient populations, ensuring a wider range of data distribution. This increased variability minimized the network's tendency to focus on localized or non‐generalizable features, ultimately contributing to a more robust and accurate model.^23^ Eight‐fold cross‐validation was used to ensure robustness and provide a reliable estimate of generalization capability. The model was trained with transfer learning on ImageNet, utilizing the Adam optimizer with a learning rate of 10^−4^ and a batch size of 5 for 50 epochs. During training, we implemented data augmentation and regularization methods to prevent overfitting and improve model performance. The model's performance was evaluated using a combination of accuracy metrics and a comprehensive set of geometric metrics. These geometric metrics captured various aspects of segmentation accuracy, including volume‐based measures (relative volume difference [RVD]), spatial overlap (dice similarity coefficient [DSC]), and spatial distance‐based measures (Hausdorff distance [HD] and average surface distance [ASD]). Quantitative assessments demonstrated a satisfactory correlation between the automatically segmented and manually outlined contours. The model generates a binary contouring mask at 224 * 224 resolution. This mask is then converted into DICOM radiotherapy structure data using a marching squares method, and up‐scaled to 512 * 512 for seamless integration into the treatment planning system (TPS). Additional post‐processing steps include 3D volumetric outlier removal and contour smoothing.

### 2.2 Patient selection

The study protocol was approved by the Research Ethics Committee of Mashhad University of Medical Sciences, Mashhad, Iran (code: IR.mums.medical.rec.1398.634). For this study, we utilized 20 planning CT volumes from a retrospective patient in the test set. Furthermore, to assess the model's robustness and generalizability, eight unseen external CT image datasets from eight localized prostate cancer patients, with different distributions from the training and testing data, were obtained from a separate radiotherapy center.

**FIGURE 1:**
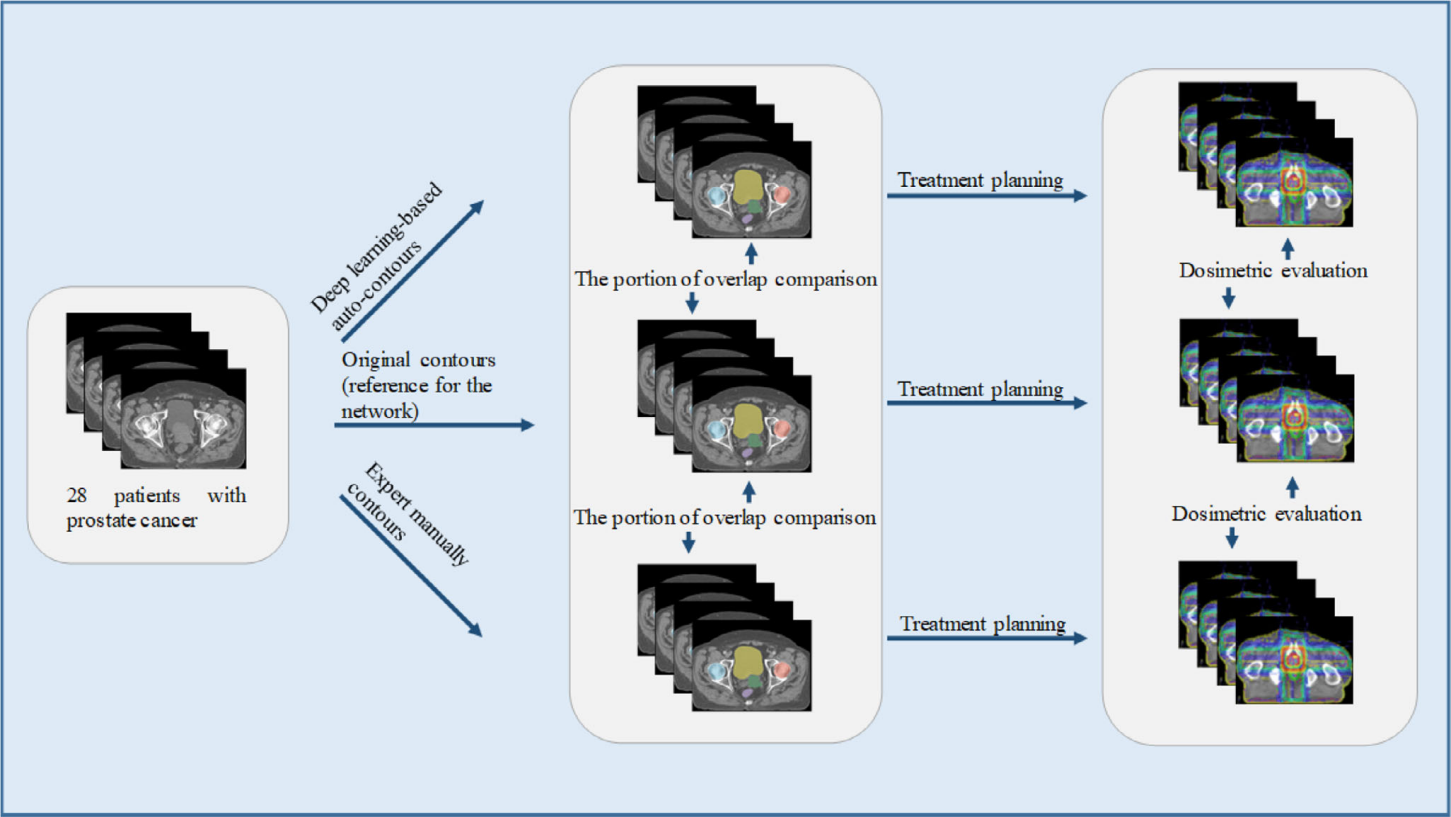
The overview of the workflow for this study.

These 28 patients received radiation therapy with a prescribed dose (PD) range of 64 to 78 Gy. Each patient had three sets of contours: the pre‐existing reference original contours (OC), the auto‐segmented contours (AC) generated by our deep learning system, and expert‐defined manual contours (EC). In our prior research, an attending radiation oncologist manually contoured the CTV and OARs, including the bladder, rectum, right femoral head (RFH), and left femoral head (LFH), for network implementation. These contours, designated as “OC,” served as the reference standard for our network. The EC were independently delineated by another highly experienced radiation oncologist on the same 28 planning CT scans, separate from the pre‐existing OC, to assess inter‐expert variability. To ensure consistent EC and OC contouring, both radiation oncologists independently reviewed the planning CT scans, incorporating detailed pathological staging and clinical information for each case to guide their evaluation and ensure accurate target and OAR delineation. Following the automatic generation of CTVs and OARs, the accuracy of these auto‐contours was assessed using spatial overlap analysis. Figure 1 shows an overview of the workflow for this study.

### 2.3 Treatment planning

Due to a high workload of intensity‐modulated radiation therapy (IMRT), we designed treatment plans for all 28 patients using 3D‐conformal radiotherapy (3D‐CRT) instead of the standard IMRT. However, considering IMRT's superior dose conformity and the requirement for more precise contouring compared to 3D‐CRT, we hypothesized that geometric discrepancies between automated and reference contours would translate into observable differences in the dose distribution (dosimetric differences) between IMRT plans generated using each contour type. To assess this impact, we created IMRT plans for five cases (three from the test set and two from external dataset).

For each patient, three treatment plans were generated for both 3D‐CRT and IMRT using the same planning CT image. These plans were based on three distinct contouring approaches: OC, AC, and EC. A consistent planning target volume (PTV) margin, determined by the radiation oncologist, was applied around the CTV for all three contour sets. This anisotropic margin varied between patients, ranging from 6 mm to 10 mm (posterior margin 5–6 mm).

### 2.4 3D‐CRT treatment planning

3D‐CRT treatment planning was performed at The Research and Treatment Center of Imam Reza Hospital, Mashhad, Iran, using the ISOgray TPS (DOSIsoft, SA, France) and an Elekta Precise linac (Elekta, Sweden) equipped with an 80‐leaf multileaf collimator (MLC) and a 15 MV x‐ray beam. The TPS utilized Collapsed‐cone to determine dose distributions. To ensure consistent dosimetric comparisons, a standardized dose of 70 Gy was applied to all 28 patients across all three contours. Conventional four‐field box radiotherapy with opposed anterior‐posterior and lateral beams was used, and treatment plans were optimized based on the corresponding contours. The dose distribution within the PTV was carefully controlled, ensuring that no “hot spots” exceeded 105% of the PD. The clinical dose criteria used are summarized in Table 1.

**TABLE 1:** Clinical objectives and dose criteria for 3D‐CRT and IMRT treatment planning.

	3D‐CRT
Volume	Dose criteria
**CTV**	100% of the CTV receives at least 100% of the prescribed dose (PD).
**PTV**	> 95% of the PTV receives at least 95% of the PD.
**Bladder**	*V*_50_ < 50% *V*_65_ < 50%
**Rectum**	*V*_50_ < 50% *V*_65 _25%
**Femoral heads**	Mean Dose < 37 Gy Max Dose < 55 Gy

	IMRT
Volume	Dose criteria
**CTV**	100% of the CTV receives 100% of the prescribed dose (PD).
**PTV**	> 90% of the PTV receives 100% of the PD. A sharp dose fall off is expected for all targets to minimize the hot dose areas.
**Bladder**	*V*_65_ < 50% *V*_70_ < 35% *V*_75_ < 25% *V*_79_ < 15%
**Rectum**	*V*_50_ < 50% *V*_60_ < 35% *V*_65_ < 20–25% *V*_70_ < 20% *V*_75_ < 15% (< 10% if possible) Max Dose < 100%–102% of the PD
Femoral heads	Mean Dose < 30–37 Gy Max Dose < 55 Gy Flexibility and Trade‐off: If achieving the maximum dose criteria for femoral heads is challenging due to constraints imposed by bladder or rectum dose limitations, the maximum dose can be increased to a maximum of 64 Gy, provided the following conditions are met: *V*_50 _< 10% *V*_55 _< 1%

Abbreviations: 3D CRT, 3D‐conformal radiotherapy; CTV, clinical target volume; IMRT, intensity‐modulated radiation therapy; PD, prescribed dose; PTV, planning target volume.

### 2.5 IMRT treatment planning

Fifteen IMRT plans were designed for five patients (three contour sets each) using a TrueBeam medical accelerator (Varian Medical Systems, Palo Alto, CA, USA) equipped with a 120 MLC at Reza Radiotherapy and Oncology Center, Mashhad, Iran. The plans were generated using the Eclipse TPS (version 16.1.2, Varian Medical Systems, Palo Alto, CA, USA) with 6 MV x‐ray and a fixed‐field dynamic intensity modulation. The IMRT plan employed a seven‐field co‐planar beam arrangement using dynamic MLC, with gantry angles of 0°, 51°, 103°, 153°, 206°, 255°, and 308°. The collimator angle was maintained at 0° for all beam configurations. Plan optimization was conducted using Photon Optimizer (version 16.1.2), and dose calculation was performed using analytical anisotropic algorithm (AAA) with a 2.5 mm grid resolution.

Identical optimization settings, objectives, and weights were applied to all three contour sets for each of the five patients to enable consistent dosimetric comparisons. The locations of the dose‐volume objectives were repeatedly adjusted during optimization to maintain a distance between the target dose and healthy tissue. Treatment planning followed standard clinical protocols, delivering a standardized dose of 78 Gy in 39 fractions to all patients across all three contour sets. The clinical objectives and dose criteria used are summarized in Table 1. Dose distributions for all patients met our clinical guidelines for the PTV, ensuring that at least 90% received the PD and that no more than 2% received a dose exceeding 105% of the PD (*D*_2%_ ≤ 105% PD).

### 2.6 Dosimetric evaluation

For each patient, we analyzed the EC, AC, and OC plans based on their dose‐volume histograms (DVHs). Plan quality and OAR sparing were evaluated using a comprehensive set of dosimetric parameters. For 3D‐CRT plans, these included *D*_98%_, *D*_95%_, *D*_2%_, *D*_max_, *D*_mean_, and *V*_50_, and *V*_65_, based on our institution's guidelines. IMRT plans were assessed using *D*_98%_, *D*_95%_, D_2%_, *V*_50_ _Gy_, *V*_65_ _Gy_, *V*_70_ _Gy_, *V*_75_ _Gy_, *V*_79_ _Gy_, *D*_max_, and *D*_mean_ based on Reza Radiotherapy and Oncology Center guidelines.

The homogeneity index (HI) and conformity index (CI) were employed to further evaluate the dosimetric characteristics of the PTV. The HI was determined using the following formula^24^:

(1) HI=D2−D98/D50

This index, with an ideal value of zero, serves as a measure of plan quality. Higher values indicate improved plan quality.

The CI of the target volume is defined as follows^25^:

(2) $$\mathrm{CI}\;=\frac{{\mathrm{TV}}_{\mathrm{RI}}}{\mathrm{TV}}\;\times \frac{{\mathrm{TV}}_{\mathrm{RI}}}{V_{\mathrm{RI}}}$$

This equation evaluates treatment quality by considering both target coverage and healthy tissue sparing. It calculates the ratio of the target volume within the reference isodose (TV_RI_) (95% of the PD) to the total target volume (TV) and the reference isodose volume (*V*_RI_). The first fraction quantifies the extent to which the target volume receives the PD, while the second fraction measures the volume of healthy tissue exposed to a dose exceeding or equal to the reference dose. The resulting CI ranges from 0 to 1, with 1 representing optimal treatment where the target is fully covered and healthy tissue exposure is minimized.

For PTV coverage and dose distribution, we further evaluated the dose distributions of PTVs derived from automatically contoured CTVs (PTV‐AC) against the ground truth contours using a 3%/3 mm 3D global gamma in both 3D‐CRT and IMRT treatment planning. Notably, gamma values were calculated only for voxels receiving at least 10% of the PD.

DVH parameters from plans based on automated contours were compared to those from plans using the OC. Furthermore, the dosimetric differences between plans based on auto‐contours and their reference contours were compared to the dosimetric differences between plans using two manually contoured sets.

### 2.7 Inter‐expert variability evaluation

In our prior work,^22^ we focused on geometrically evaluating model performance from various perspectives. In the present study, to assess the network's performance relative to inter‐observer variability, we calculated the DSC, a measure of spatial overlap, for AC‐OC, AC‐EC, and EC‐OC pairs. This analysis compared the overlap between two manual contours (EC‐OC) with the overlap between automated and their original manual reference contours (AC‐OC), and with the overlap between automated and second set of manual contours (AC‐EC). A higher DSC value indicates greater overlap, calculated as:

(3) $$\begin{array}{ccc} \mathrm{DSC}\; & = & 2*\left(\mathrm{Volume}\;1\cap \mathrm{Volume}\;2\right)/\\ & & \left(\mathrm{Volume}\;1+\mathrm{Volume}\;2\right) \end{array}$$

A two‐sided Mann‐Whitney *U* test with a significance threshold of 0.05 was used to determine if the differences between network results and inter‐expert variability were statistically significant. Statistical analysis was performed using SPSS version 19.0.

## 3 RESULTS

### 3.1 Inter‐expert variability results

**FIGURE 2:**
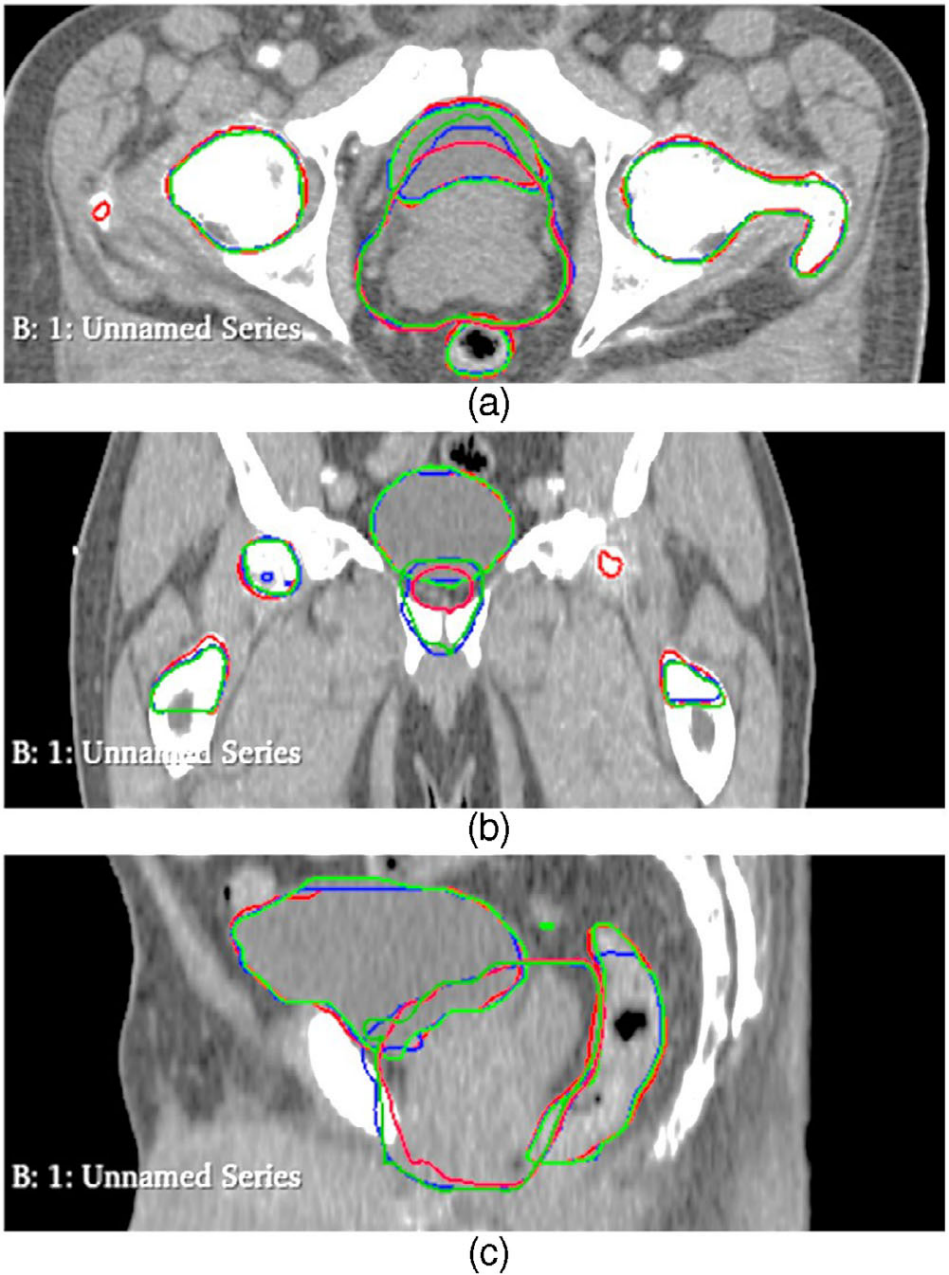
The three different contours (EC, AC, and OC) for a typical patient in axial (a), sagittal (b), and coronal (c) views. Red lines represent AC, blue lines represent OC, and green lines represent EC. AC, auto‐segmented contours; EC, expert‐defined manual contours; OC, reference original contours.

Table 2 presents a comparison of DSC for automated and manually generated contours. The table indicates that comparing automated contours (AC) to their original manual reference contours (OC) yields higher DSC values than comparing two sets of manually generated contours (EC‐OC). This observation holds true for all anatomical volumes except the rectum. Furthermore, comparing AC to a second set of manual contours (EC) generally results in higher DSC values than comparing two sets of manual contours (EC‐OC), with the exception of the prostate and rectum. Figure 2 presents the three different contours (EC, AC, and OC) for a typical patient in axial, sagittal, and coronal views.

**TABLE 2:** Summary of DSC for CTV and OARs (Mean ± SD).

	Testing set and unseen external data (28 patients)				
	DSC			*p*‐value	*p*‐value
Volume	EC‐OC	AC‐OC	AC‐EC	EC‐OC vs. AC‐OC	EC‐OC vs. AC‐EC
**CTV**	90.04 ± 1.24	91.18 ± 1.65	89.85 ± 1.18	0.10	0.08
**Bladder**	93.20 ± 1.11	95.46 ± 0.84	95.40 ± 0.67	0.25	0.36
**Rectum**	91.60 ± 2.62	87.52 ± 1.72	86.81 ± 2.11	0.02*	0.03*
**RFH**	94.45 ± 0.56	96.50 ± 0.80	95.63 ± 0.29	0.09	0.18
**LFH**	94.69 ± 0.38	96.67 ± 0.65	95.20 ± 0.89	0.08	0.21

*Note*: Statistically significant differences (p<0.05) between AC‐EC and EC‐OC, as well as AC‐OC and EC‐OC, were observed for the rectum.

Abbreviations: AC, auto‐segmented contours; CTV, clinical target volume; DSC, dice similarity coefficient; EC, expert‐defined manual contours; OAR, organs at risk; OC, reference original contours.

### 3.2 Clinical dosimetric evaluation

DVH comparisons of the PTV and OARs between the EC plan, AC plan, and OC plan revealed an acceptable agreement for each patient. Tables 3, 4, and 5 present the mean and standard deviation (SD) of dosimetric indices for 3D‐CRT (20 cases), 3D‐CRT (external set of eight cases), and IMRT (five cases), respectively. Statistical analysis was conducted for further evaluation. To statistically compare groups, we employed the Wilcoxon signed‐rank test, considering a p‐value below 0.05 as statistically significant.

**TABLE 3:** Quantitative dosimetric evaluation for 3D CRT plans of 20 patients for the three different contour sets.

	3D CRT plans of testing set							
Volume	Dosimetric index	EC Mean ± SD	OC Mean ± SD	AC Mean ± SD	Δ│ EC‐OC│ Mean ± SD	Δ│ AC‐OC│ Mean ± SD	*p*‐value EC‐OC	*p*‐value AC‐OC
**PTV**	*D*_98%_ /Gy *D*_95%_ /Gy *D*_2%_ /Gy *D*_max_ /Gy *D*_mean_ /Gy HI CI	65.90 ± 0.21 67.02 ± 0.58 70.02 ± 0.09 70.76 ± 0.69 69.07 ± 0.37 0.09 ± 0.02 0.63 ± 0.02	66.05 ± 0.48 66.61 ± 0.41 70.02 ± 0.14 70.81 ± 0.80 68.70 ± 0.89 0.08 ± 0.02 0.68 ± 0.02	66.17 ± 0.11 66.79 ± 0.38 70.03 ± 0.51 70.80 ± 0.89 68.53 ± 1.33 0.07 ± 0.03 0.65 ± 0.02	0.15 ± 0.52 0.41 ± 0.71 0.00 ± 0.17 0.05 ± 1.06 0.37 ± 0.97 0.01 ± 0.03 0.05 ± 0.03	0.12 ± 0.49 0.18 ± 0.56 0.01 ± 0.53 0.01 ± 1.22 0.17 ± 1.60 0.01 ± 0.04 0.03 ± 0.03	0.22 0.14 0.35 0.47 0.29 0.51 0.40	0.19 0.29 0.34 0.52 0.55 0.32 0.32
**Bladder**	*V*_50_ _Gy_ /% *V*_65_ _Gy_ /% *D*_max_ /Gy *D*_mean_ /Gy	31.29 ± 16.03 28.37 ± 9.14 69.43 ± 1.30 35.05 ± 15.24	29.50 ± 20.33 27.22 ± 15.03 69.41 ± 0.97 30.77 ± 16.06	28.57 ± 22.56 26.05 ± 8.09 69.15 ± 0.79 29.08 ± 15.49	1.79 ± 26.14 1.15 ± 16.00 0.02 ± 1.46 4.28 ± 10.05	0.93 ± 30.23 1.17 ± 17.04 0.26 ± 1.27 1.69 ± 22.38	0.34 0.11 0.41 0.06	0.20 0.16 0.57 0.24
**Rectum**	*V*_50_ _Gy_ /% *V*_65_ _Gy_ /% *D*_max_ /Gy *D*_mean_ /Gy	32.25 ± 13.80 20.89 ± 8.02 69.41 ± 0.17 37.90 ± 8.20	35.26 ± 16.60 24.31 ± 3.44 69.55 ± 0.70 37.62 ± 12.93	37.07 ± 15.02 22.87 ± 11.00 69.66 ± 0.42 33.86 ± 11.05	3.01 ± 21.57 3.42 ± 8.78 0.14 ± 0.73 0.28 ± 15.35	1.81 ± 22.29 1.44 ± 11.56 0.11 ± 0.82 3.76 ± 17.26	0.07 0.05 0.22 0.33	0.11 0.08 0.22 0.16
**RFH**	*D*_max_ /Gy *D*_mean_ /Gy	46.27 ± 4.42 30.21 ± 4.11	45.90 ± 5.13 27.81 ± 3.77	46.14 ± 2.98 26.52 ± 4.94	0.37 ± 6.82 2.40 ± 6.96	0.24 ± 6.02 1.29 ± 6.28	0.13 0.09	0.18 0.24
LFH	*D*_max_ /Gy *D*_mean_ /Gy	44.76 ± 1.02 29.55 ± 6.59	46.77 ± 3.28 26.88 ± 5.97	45.62 ± 3.19 25.45 ± 6.91	2.01 ± 3.44 2.67 ± 8.86	1.15 ± 4.56 1.43 ± 9.08	0.18 0.04*	0.10 0.25

**Abbreviations**: 3D CRT, 3D conformal radiotherapy; AC, auto‐segmented contours; EC, expert‐defined manual contours; HI, Homogeneity index.; LFH, left femoral head; OC, reference original contours; PTV, planning target volume; RFH, right femoral head.

**TABLE 4:** Quantitative dosimetric evaluation for 3D CRT plans of eight external patients for the three different contour sets.

	3D CRT plans of eight external patients							
Volume	Dosimetric index	EC Mean ± SD	OC Mean ± SD	AC Mean ± SD	Δ│ EC‐OC│ Mean ± SD	Δ│ AC‐OC│ Mean ± SD	*p*‐value EC‐OC	*p*‐value AC‐OC
**PTV**	*D*_98%_ /Gy *D*_95%_ /Gy *D*_2%_ /Gy *D*_max_ /Gy *D*_mean_ /Gy HI CI	65.66 ± 0.15 66.84 ± 0.34 70.62 ± 0.22 70.91 ± 0.30 69.11 ± 0.31 0.11 ± 0.06 0.61 ± 0.10	65.94 ± 0.44 67.09 ± 0.44 70.40 ± 0.13 70.61 ± 0.91 69.07 ± 0.21 0.09 ± 0.04 0.59 ± 0.15	66.05 ± 0.16 66.12 ± 0.60 70.19 ± 0.49 70.04 ± 1.01 69.19 ± 0.87 0.07 ± 0.08 0.63 ± 0.23	0.28 ± 0.46 0.25 ± 0.54 0.22 ± 0.32 0.30 ± 0.98 0.04 ± 0.47 0.02 ± 0.07 0.02 ± 0.18	0.11 ± 0.47 0.97 ± 0.70 0.21 ± 0.51 0.57 ± 1.52 0.12 ± 0.90 0.02 ± 0.09 0.04 ± 0.26	0.22 0.16 0.13 0.33 0.11 0.41 0.57	0.18 0.24 0.26 0.25 0.21 0.39 0.35
**Bladder**	*V*_50_ _Gy_ /% *V*_65_ _Gy_ /% *D*_max_ /Gy *D*_mean_ /Gy	35.41 ± 5.22 31.75 ± 4.14 68.22 ± 1.30 35.38 ± 7.46	34.04 ± 1.63 29.67 ± 3.91 68.74 ± 2.91 36.28 ± 8.92	33.06 ± 3.50 30.96 ± 2.24 69.29 ± 0.99 37.23 ± 4.33	1.37 ± 4.66 2.08 ± 5.27 0.52 ± 2.76 0.90 ± 9.65	0.98 ± 3.84 1.29 ± 4.30 0.55 ± 2.66 0.95 ± 7.82	0.21 0.09 0.13 0.46	0.25 0.27 0.26 0.32
**Rectum**	*V*_50_ _Gy_ /% *V*_65_ _Gy_ /% *D*_max_ /Gy *D*_mean_ /Gy	38.63 ± 7.55 26.51 ± 3.88 69.34 ± 1.02 39.68 ± 3.41	40.39 ± 8.54 24.23 ± 5.40 69.52 ± 1.67 38.51 ± 1.86	44.83 ± 9.21 30.61 ± 6.85 70.62 ± 2.48 42.69 ± 6.89	1.76 ± 11.32 2.28 ± 6.59 0.18 ± 1.48 1.17 ± 3.37	4.44 ± 12.63 6.38 ± 8.72 1.10 ± 2.97 4.18 ± 7.12	0.15 0.10 0.56 0.23	0.04* 0.02* 0.21 0.04*
**RFH**	*D*_max_ /Gy *D*_mean_ /Gy	43.35 ± 2.48 28.12 ± 1.79	40.99 ± 5.19 29.73 ± 3.71	43.19 ± 1.33 28.45 ± 1.98	2.36 ± 5.61 1.61 ± 4.03	2.20 ± 5.38 1.28 ± 4.07	0.15 0.32	0.21 0.19
LFH	*D*_max_ /Gy *D*_mean_ /Gy	42.71 ± 0.84 26.49 ± 3.65	43.72 ± 1.32 26.81 ± 1.01	41.74 ± 2.12 27.58 ± 3.83	1.01 ± 1.62 0.32 ± 3.71	1.98 ± 2.85 0.77 ± 4.15	0.17 0.44	0.22 0.48

Abbreviations: 3D CRT, 3D conformal radiotherapy; AC, auto‐segmented contours; EC, expert‐defined manual contours; HI, Homogeneity index.; LFH, left femoral head; OC, reference original contours; PTV, planning target volume; RFH, right femoral head.

**TABLE 5:** Quantitative dosimetric evaluation for IMRT plans of five patients for the three different contour sets.

	IMRT plans of five patients							
Volume	Dosimetric index	EC Mean ± SD	OC Mean ± SD	AC Mean ± SD	Δ│ EC‐OC│ Mean ± SD	Δ│ AC‐OC│ Mean ± SD	*p*‐value EC‐OC	*p*‐value AC‐OC
**PTV**	*D*_98%_ /Gy *D*_95%_ /Gy *D*_2%_ /Gy *V*_78_ _Gy_ /% *D*_mean_/Gy *D*_max_/Gy HI CI	76.78 ± 0.14 77.71 ± 0.53 81.12 ± 0.32 93.52 ± 2.58 78.57 ± 0.52 81.54 ± 0.37 0.05 ± 0.01 0.90 ± 0.10	76.31 ± 0.88 77.58 ± 0.69 81.05 ± 1.04 91.74 ± 1.41 78.37 ± 0.80 82.78 ± 0.24 0.04 ± 0.03 0.91 ± 0.06	76.75 ± 0.29 77.62 ± 0.38 81.13 ± 0.92 93.86 ± 2.38 78.54 ± 0.69 82.14 ± 0.91 0.05 ± 0.03 0.89 ± 0.01	0.47 ± 0.89 0.13 ± 0.87 0.07 ± 1.09 1.78 ± 2.92 0.20 ± 0.95 1.24 ± 0.44 0.01 ± 0.03 0.01 ± 0.12	0.44 ± 0.94 0.04 ± 0.80 0.08 ± 1.40 2.12 ± 2.77 0.17 ± 1.05 0.64 ± 0.94 0.01 ± 0.04 0.02 ± 0.06	0.09 0.16 0.41 0.11 0.31 0.14 0.36 0.10	0.14 0.24 0.38 0.20 0.30 0.34 0.51 0.06
**Bladder**	*V*_65_ _Gy_ /% *V*_70_ _Gy_ /% *V*_75_ _Gy_ /% *V*_79_ _Gy_ /% *D*_mean_ /Gy	20.57 ± 0.56 17.24 ± 0.44 15.37 ± 0.06 13.91 ± 0.03 12.40 ± 0.22	23.94 ± 1.86 16.44 ± 1.01 14.40 ± 0.94 13.89 ± 0.03 15.71 ± 0.38	20.66 ± 1.21 17.64 ± 0.76 16.19 ± 0.58 14.81 ± 0.03 14.11 ± 0.92	3.37 ± 1.95 0.80 ± 1.10 0.97 ± 0.94 0.02 ± 0.04 3.31 ± 0.44	3.28 ± 2.19 1.20 ± 1.28 1.79 ± 1.10 0.92 ± 0.04 1.60 ± 1.00	0.08 0.21 0.30 0.41 0.04*	0.14 0.18 0.12 0.31 0.08
**Rectum**	*V*_50_ _Gy_ /% *V*_60_ _Gy_ /% *V*_65_ _Gy_ /% *V*_70_ _Gy_ /% *V*_75_ _Gy_ /% *D*_max_/Gy *D*_mean_/Gy	28.50 ± 2.80 25.86 ± 2.11 16.91 ± 1.78 10.40 ± 1.74 10.28 ± 0.96 81.29 ± 0.77 28.14 ± 0.89	30.96 ± 3.14 27.62 ± 1.39 16.80 ± 2.20 13.83 ± 0.97 10.41 ± 1.01 81.48 ± 0.21 32.79 ± 1.52	29.07 ± 1.22 22.90 ± 3.29 13.24 ± 1.11 14.02 ± 1.16 10.76 ± 1.31 81.50 ± 0.40 30.71 ± 1.24	2.46 ± 4.22 1.76 ± 2.53 0.11 ± 2.85 3.43 ± 2.00 0.13 ± 1.41 0.19 ± 0.80 4.65 ± 1.77	1.89 ± 3.38 4.72 ± 3.56 3.56 ± 2.47 0.19 ± 1.53 0.35 ± 1.65 0.02 ± 0.45 2.08 ± 1.99	0.13 0.12 0.23 0.06 0.25 0.31 0.06	0.18 0.03* 0.05 0.17 0.19 0.54 0.09
**RFH**	*D*_max_/Gy *D*_mean_/Gy	33.61 ± 1.92 6.52 ± 0.93	35.19 ± 1.01 6.37 ± 0.88	33.38 ± 3.24 6.21 ± 1.14	1.58 ± 2.15 0.15 ± 1.31	1.81 ± 3.39 0.16 ± 1.47	0.16 0.43	0.20 0.31
LFH	*D*_max_/Gy *D*_mean_/Gy	35.15 ± 1.59 5.74 ± 0.89	35.12 ± 2.79 5.65 ± 1.09	34.08 ± 0.91 5.54 ± 0.87	0.03 ± 3.22 0.09 ± 1.42	1.04 ± 2.92 0.11 ± 1.41	0.61 0.52	0.29 0.35

Abbreviations: AC, auto‐segmented contours; EC, expert‐defined manual contours; HI, Homogeneity index.;IMRT, intensity‐modulated radiation therapy; LFH, left femoral head; OC, reference original contours; PTV, planning target volume; RFH, right femoral head.

### 3.3 Clinical evaluation of 3D‐CRT plans

All 3D‐CRT plans successfully met clinical needs for PTV coverage. In the test set of 20 patients, the average *D*_95_ for PTV was 67.02 ± 0.58 Gy for EC plans, 66.61 ± 0.41 Gy for OC contours, and 66.79 ± 0.38 Gy for AC plans. For the eight external patients, the average *D*_95_ values were 66.84 ± 0.34 Gy, and 67.09 ± 0.44 Gy, and 66.12 ± 0.60 Gy for EC, OC, and AC plans, respectively. The *D*_95_ achieved using PTVs derived from automatically contoured CTVs (PTV‐AC) demonstrated high consistency with the *D*_95_ achieved using reference PTV contours (PTV‐OC) for all cases, indicating accurate target coverage. Similarly, *D*_95_ showed agreement between plans based on two manual PTV contours (PTV‐EC and PTV‐OC).

**FIGURE 3:**
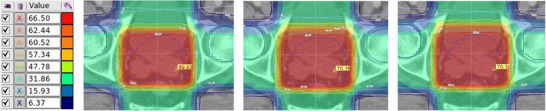
Axial plane dose distributions in color wash for an exemplary patient across three plans: AC‐plan (left), OC‐plan (middle), and EC‐plan (right). The plans generated using four‐field box radiotherapy 3D‐CRT aiming for a PD of 70 Gy. The red line represents the 95% isodose line coverage of the PTV (66.5 Gy). 3D CRT, 3D‐conformal radiotherapy; AC, auto‐segmented contours; EC, expert‐defined manual contours; OC, reference original contours; PD, prescribed dose; PTV, planning target volume.

All 3D‐CRT plans achieved a *D*_2%_ value closer to the PD, with a *D*_2%_ value of 105% or less for the PTV. The average HI for the PTV across 20 patients in the test set was 0.09 ± 0.02 for EC plans, 0.08 ± 0.02 for OC plans, and 0.07 ± 0.03 for AC plans. Treatment plans of eight external patients yielded average HI values of 0.11 ± 0.06 for EC plans, 0.09 ± 0.04 for OC plans, and 0.07 ± 0.08 for AC plans. Figure 3 shows axial plane of dose distributions for an exemplary patient planned with 3D‐CRT, comparing AC, OC, and EC plans.

3D global gamma analysis of PTVs derived from automatically contoured CTVs of 20 patients and compared to ground truth contours, demonstrated pass rates ranging from 91.21% to 96.82%, averaging 95.55% for 3D‐CRT treatment planning. For plans of eight external patients, the pass rates ranged from 90.82% to 95.90%, with an average of 93.31%.

DVH analysis, performed on both a test set of 20 patients and eight external patients, demonstrated consistent dose distributions for the bladder and rectum across three different contour sets. All rectal and bladder dose‐volume constraints met clinical requirements. The volume receiving 50 Gy (*V*_50_) remained below 40% for the bladder and 50% for the rectum, except for one patient whose rectal *V*_50_ exceeded 50% across all three contour sets.

**FIGURE 4:**
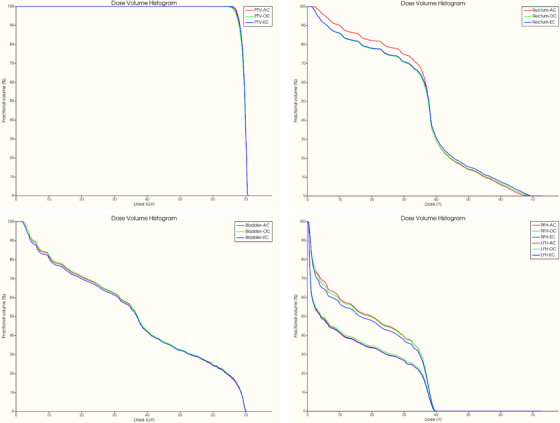
DVHs for a typical patient planned with 3D‐CRT, illustrating the dose distribution to the PTV and OARs, including the rectum, bladder, and femoral heads. 3D CRT, 3D‐conformal radiotherapy; DVH, dose‐volume histograms; OAR, organs at risk; PTV, planning target volume.

Additionally, the average dose (*D*_mean_) delivered to the femoral heads remained below 35 Gy across three different contour sets, for both a test set of 20 patients and eight external patients. Figure 4 shows the DVHs for a typical patient planned with 3D‐CRT, illustrating the dose distribution to the PTV and OARs including the rectum, bladder, and femoral heads.

### 3.4 Clinical Evaluation of IMRT Plans

All fifteen IMRT plans successfully met clinical needs. The average *D*_95_ for the PTV across five patients was 77.71 ± 0.53 Gy for EC plans, 77.58 ± 0.69 Gy for OC plans, and 77.62 ± 0.38 Gy for AC plans, indicating accurate target coverage across all cases. The average PTV volume achieving 100% of the PD (*V*_78_ _Gy_) across five patients was 93.52 ± 2.58 % for EC plans, 91.74 ± 1.41 % for OC plans, and 93.86 ± 2.38 % for AC plans.

**FIGURE 5:**
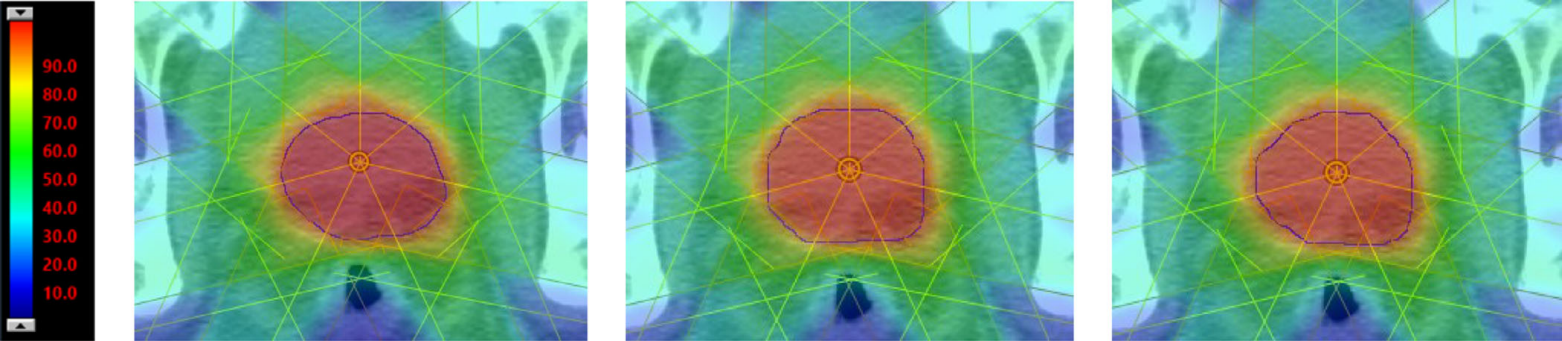
Axial plane dose distributions in color wash for an exemplary patient across three plans: AC‐plan (left), OC‐plan (middle), and EC‐plan (right). The plans generated using seven‐field IMRT aiming for a PD of 78 Gy. AC, auto‐segmented contours; EC, expert‐defined manual contours; IMRT, intensity‐modulated radiation therapy; OC, reference original contours; PD, prescribed dose.

All fifteen plans achieved a *D*_2%_ value of 105% or less of the PD for the PTV. Across five patients, the HI for the PTV was 0.05 ± 0.01 for EC plans, 0.04 ± 0.03 for OC plans, and 0.05 ± 0.03 for AC plans. The CI for the PTV was 0.90 ± 0.10 for EC plans, 0.91 ± 0.06 for OC plans, and 0.89 ± 0.01 for AC plans. Figure 5 shows axial plane of dose distributions for an exemplary patient planned with IMRT, comparing AC, OC, and EC plans.

A 3D global gamma analysis of five patients, comparing automatically contoured CTVs to ground truth contours for IMRT plans, yielded pass rates ranging from 93.45% to 95.06%, with an average of 94.13%.

**FIGURE 6:**
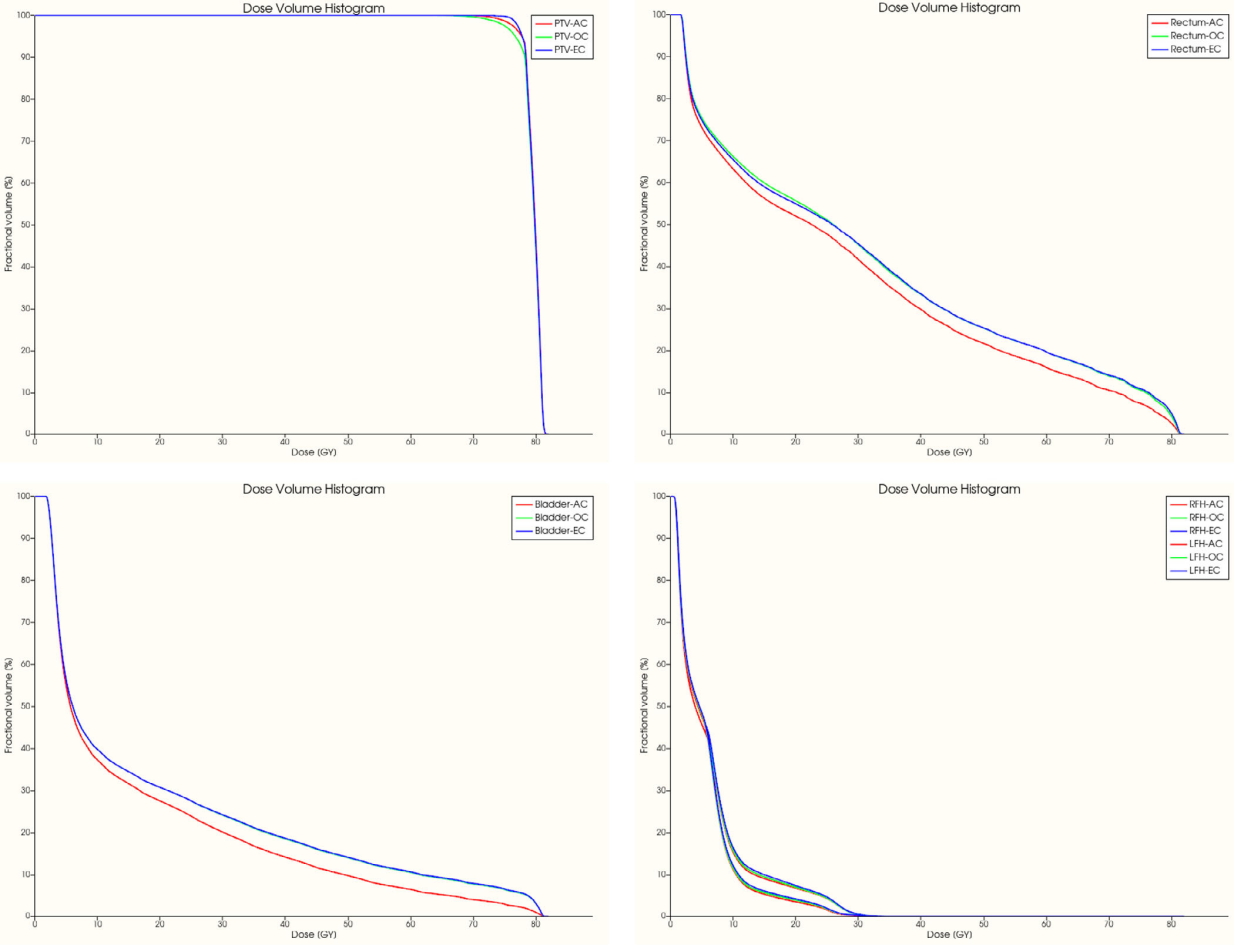
DVHs for a typical patient planned with IMRT, illustrating the dose distribution to the PTV and OARs, including the rectum, bladder, and femoral heads. DVH, dose‐volume histograms; IMRT, intensity‐modulated radiation therapy; OAR, organs at risk; PTV, planning target volume.

DVH analysis, performed on five patients, demonstrated consistent dose distributions for the bladder, rectum, and femoral heads across three different contour sets. All rectal dose‐volume constraints, including *V*_50_ _Gy_, *V*_60_ _Gy_, *V*_65_ _Gy_, *V*_70_ _Gy_, and *V*_75_ _Gy_, met our center's guidelines, with V_50_ remaining well below 50%. Similarly, all bladder dose‐volume constraints, including *V*_65_ _Gy_, *V*_70_ _Gy_, *V*_75_ _Gy_, and *V*_79_ _Gy_, met clinical requirements. The average (*D*_mean_) and maximum (*D*_max_) doses to the femoral heads remained well below 30 Gy and 55 Gy, respectively, across all contour sets. Figure 6 shows the DVHs for a typical patient planned with IMRT, illustrating the dose distribution to the PTV and OARs including the rectum, bladder, and femoral heads.

### 3.5 Statistical evaluation

#### 3.5.1 Statistical evaluation of 3D‐CRT plans

For 3D‐CRT plans of eight external patients, no statistically significant dosimetric differences were observed between automated and manual contours for the PTV and OARs, except for the rectum (*V*_50_ _Gy_, *V*_65_ _Gy_, and *D*_mean_). In the testing set, a statistically significant difference was detected in the mean dose (*D*_mean_) to the LFH when comparing the two manual contouring sets.

#### 3.5.2 Statistical evaluation of IMRT plans

In IMRT plans, no significant dosimetric differences were found between automated and manual contours for the PTV and OARs, except for the volume receiving 60 Gy (*V*_60_ _Gy_) for the rectum. Similarly, the two manual contouring sets demonstrated no significant dosimetric differences, with the exception of the mean dose (*D*_mean_) to the bladder.

#### 3.5.3 Contouring time

**FIGURE 7:**
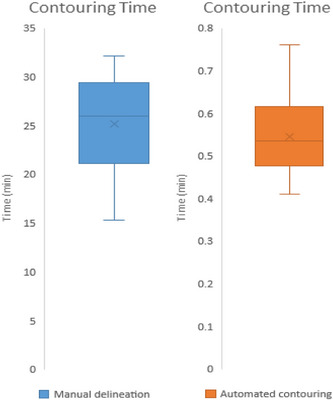
Distribution of contouring times for manual and automated contouring of the prostate region.

Contouring times were compared for manual and automated methods using data from 22 patients: eight from an external dataset and 14 from testing set. Six patients in the testing set were excluded due to missing contouring times. Contouring time for each patient represents the total time spent contouring, measured from the start of the first contour to the completion of the last contour. Manual delineation, utilizing the ISOgray TPS system with mesh‐based 3D reconstruction, averaged 24.9 ± 4.5 min (range: 15.3–32.1 min). Conversely, automated contouring, generated on a GPU‐based system (1.6 GHz GPU frequency, 8 GB VRAM), averaged 0.53 ± 0.08 min (range: 0.41–0.76 min). Figure 7 displays a box plot illustrating the distribution of contouring time for each method.

## 4 DISCUSSION

Our previous research demonstrated the promising geometric accuracy of the deep‐learning‐based auto‐segmentation contour model for CTV and OARs in radiotherapy of prostate cancer patients. We achieved a satisfactory geometric correlation between automated and manually outlined contours. This study further investigates the dosimetric performance of these auto‐contours using standard metrics like DVH, *D*_98%_, *D*_95%_, *D*_2%_, and *D*_mean_, providing insight into their accuracy, reliability, and potential clinical impact.

To ensure a robust evaluation, our approach closely resembles real‐world clinical practice, aligning with methods proposed in other research.^26^, ^27^ The DSCs obtained between two sets of manual contours (EC‐OC) are consistent with results reported in previous studies.^28^, ^29^

Table 2 shows that the geometric differences between automated contours (AC) and both their original manual reference contours (OC) and a second set of manually generated contours (EC) are smaller than the differences between two manually contoured sets for bladder, RFH, and LFH structures. This finding suggests that the automated contouring system can reduce inter‐expert variability. These findings align with Wong et al.’s^30^ observations, which showed that geometric differences between automated and reference contours are smaller than those between two manually contours for bladder and femoral heads.

Furthermore, the auto‐contouring system demonstrated satisfactory dosimetric performance in both 3D‐CRT and IMRT plans.  The small geometic difference between automatic and reference segmentations did not lead to dosimetric differences exceeding random variations. PTV coverage and dose distribution were evaluated using various dosimetric parameters (*D*_98%_, *D*_95%_, *D*_2%_, *D*_max_, *D*_mean_, *V*_50 Gy_, *V*_65 Gy_, HI, CI, and gamma pass rate), and all met our clinical guidelines for all three sets of contours.

This consistency in dose distribution accuracy, using PTVs derived from automatically contoured CTVs and OARs, was confirmed across all results. Our study achieved optimal target coverage and gamma pass rate for PTVs derived from automatically contoured CTVs in both 3D‐CRT and IMRT plans. This improvement over both Starke et al.^17^ and Kawula et al.’s^2^ findings, which reported suboptimal target coverage and discrepancies in dose distribution near target boundaries, may be attributed to the greater similarity between our automatically generated prostate CTV contours and ground truth contours (DSC 91.18%) compared to their studies (DSC of 85% and 87%, respectively). As Van Dijk et al. found that auto‐contours with higher precision resulted in smaller dosimetric differences compared to manual contours.^16^

IMRT plans met all clinical dose‐volume constraints for the rectum, bladder, and femoral heads. While OAR doses were generally acceptable in all 3D‐CRT plans, except for one patient who exhibited a rectal *V*_50_ _Gy_ exceeding 50% across all three contour sets. This patient displayed significant rectal distension and a concave posterior prostatic border on the planning CT images. IMRT was used for the actual treatment delivery to address this complex case.

In the 3D‐CRT plans of testing set, the mean dose (*D*_mean_) to the LFH differed significantly between two manual contouring methods. Similarly, for the rectum in 3D‐CRT plans of eight external patients, *V*_50_ _Gy_, *V*_65_ _Gy_ and *D*_mean_ showed significant variations between automated and reference contours. IMRT plans also exhibited significant differences in rectal *V*_60_ _Gy_ and *V*_65_ _Gy_ when comparing automated contours to reference contours, as well as in bladder mean dose between two manual contours. Notably, despite these statistically significant variations, all dose levels remained within clinically acceptable ranges.

Our results demonstrate that automatic contouring had no significant impact on clinically relevant dosimetric parameters in both 3D‐CRT and IMRT treatment plans. This suggests that even for IMRT plans, which demand more precise contouring, automatic contouring does not compromise treatment planning accuracy.

Additionally, Automated contouring of the prostate region resulted in a 97.8% time reduction (24.3 min faster) compared to manual contouring. It's important to note that import and export time for image or structure files is not factored into the automated contouring times.

To maintain consistency with our network analysis, we used manually generated contours as the reference, instead of manually adjusted automated contours. While we acknowledge the potential for manual adjustments to automated contours and the impact on time savings, this manuscript compares the time efficiency of form‐scratch manual contouring (which served as the gold standard for our analysis) with automated methods. Further investigation is needed to assess the impact of these adjustments on the time savings achieved through automated contouring.

This study has some limitations. While all cases utilized 3D‐CRT, IMRT treatment planning was only performed for five patients due to a high IMRT workload. Additionally, the inter‐expert variability analysis was limited by its consideration of only two experts, resulting in less accurate estimations of overall inter‐expert variability. Finally, the dosimetric analysis relied on static treatment plans, neglecting geometric uncertainties arising from patient movement and setup variations.^31^, ^32^

## 5 CONCLUSION

This paper highlighted the feasibility of employing deep learning‐based auto‐segmentation for CTV and OAR contouring in prostate cancer radiotherapy treatment planning. The automated contouring system can reduce inter‐expert variability and achieve dosimetric accuracy comparable to gold standard reference contours, highlighting its potential for streamlining clinical workflows. Quantitative analysis revealed no consistent trend of increasing or decreasing PTVs derived from automatically contoured CTVs and OAR doses due to automated contours, indicating minimal impact on treatment outcomes. Notably, the study demonstrated a significant time reduction when using automatically generated contours compared to manual contouring. Participating radiation oncologists found the automated contours to be usable as starting templates, enabling time savings by eliminating the need for from‐scratch manual contouring.

## AUTHOR CONTRIBUTIONS

Najmeh Arjmandi led the comprehensive development and execution of this project, encompassing data collection, image processing, treatment planning, analysis, and manuscript writing. Mohammad Amin Mosleh‐Shirazi provided valuable scientific advice, contributed to the analysis of the results. Shokoufeh Mohebbi approved the IMRT treatment plans, while Shahrokh Nasseri and Alireza Mehdizadeh contributed to the final version of the manuscript. Sare Hosseini and Zohreh Pishevar contributed to organ contouring on the TPS system. Amin Amiri Tehranizadeh contributed to data collection and manuscript writing. Mehdi Momennezhad served as the project supervisor. All authors collaborated on interpreting the results and providing feedback on the manuscript.

## CONFLICT OF INTEREST STATEMENT

The authors declare no conflicts of interest.

## ETHICS APPROVAL

This study was reviewed and approved by the Ethics Committee of Mashhad University of Medical Sciences, with approval reference number IR.MUMS.MEDICAL.REC.1399.667.

## Data Availability

The datasets used and analyzed in this study are available from the corresponding author upon reasonable request.
